# Acetaminophen Use for Fever in Children Associated with Autism Spectrum Disorder

**DOI:** 10.4172/2165-7890.1000170

**Published:** 2016-03-28

**Authors:** Stephen T Schultz, Georgianna G Gould

**Affiliations:** Department of Physiology, School of Medicine, University of Texas Health Science Center San Antonio, 7703 Floyd Curl Drive, San Antonio, TX 78229, USA

**Keywords:** Autism, Autism spectrum disorder, Acetaminophen, Anandamide, Endocannabinoid, Cannabinoid, Fever, Medication

## Abstract

Autism Spectrum Disorder (ASD) is characterized by persistent deficits in social communication and restrictive behavior, interests, and activities. Our previous case-control study showed that use of acetaminophen at age 12–18 months is associated with increased likelihood for ASD (OR 8.37, 95% CI 2.08–33.7). In this study, we again show that acetaminophen use is associated with ASD (p = 0.013). Because these children are older than in our first study, the association is reversed; fewer children with ASD vs. non-ASD children use acetaminophen as a “first choice” compared to “never use” (OR 0.165, 95% CI 0.045, 0.599). We found significantly more children with ASD vs. non- ASD children change to the use of ibuprofen when acetaminophen is not effective at reducing fever (p = 0.033) and theorize this change in use is due to endocannabinoid system dysfunction. We also found that children with ASD vs. non-ASD children are significantly more likely to show an increase in sociability when they have a fever (p = 0.037) and theorize that this increase is due to anandamide activation of the endocannabinoid system in ASD children with low endocannabinoid tone from early acetaminophen use. In light of this we recommend that acetaminophen use be reviewed for safety in children.

## Introduction

Autism Spectrum Disorder (ASD), as defined by the Diagnostic and Treatment Manual for Mental Disorders, Fifth Edition (DSM-5), is characterized by persistent deficits in social communication and interaction and restricted-repetitive patterns of behavior, interests, or activities. These symptoms manifest in early childhood, and produce clinically significant developmental impairment [1]. Many children with ASD share traits of Attention Deficit/hyperactivity disorder (ADHD) and epilepsy is comorbid with ASD in an estimated 20–25% of cases [1–4]. Some features of ASD, commonly called autism or autistic disorder, are seen in genetic and chromosomal abnormalities such as fragile X syndrome, Down syndrome, and genomic insertions and deletions; however, most cases of ASD have an unknown etiology. Two of the prominent clinical features of ASD are immune system dysregulation and abnormal brain connectivity [5–7]. The US Centers for Disease Control and Prevention (CDC) estimates that ASD occurs in one of every 68 children in the US, while the National Health Interview Survey puts the estimate of ASD higher with one child affected out of every 45 children aged 3–17 years in the US [8,9].

Recently it has been shown that more than half of ASD cases are attributable to environmental factors [10]. A subset of children with ASD undergo a period of apparently normal development followed by a regression in development [11]. Since children with regression in development did not initially manifest ASD features, they may have been more likely influenced by drugs or other environmental exposures. In our own data, regression was featured in 38% of cases, and we were able to show an increased likelihood for ASD from acetaminophen use, which was higher in children with regression [12]. Prenatal and perinatal use of acetaminophen, also known as paracetamol, was linked to ASD in an ecological study [13]. We have theorized that acetaminophen disruption of the endocannabinoid system may underlie some of the increased likelihood for ASD, particularly in children with genetically compromised primary conjugation pathways that normally metabolize this drug [14].

Acetaminophen is one of three analgesics derived from aniline dye; the others, acetanilide and phenacetin, have been discontinued due to side effects [15]. Although acetaminophen has been used as an analgesic for more than a hundred years, its mechanism of action was unclear. It has now been shown that acetaminophen produces analgesia by acting in the brain as an indirect agonist at cannabinoid receptors through conversion of the acetaminophen metabolite p-aminophenol to N-arachidonoylaminophenol (AM404) which inhibits the reuptake of anandamide [15–17]. Blocking cannabinoid receptors completely eliminates the analgesic effect of acetaminophen [15,18].

We have shown in a case-control study that use of acetaminophen early in life is associated with increased likelihood for ASD [12]. We showed in this study that children who used acetaminophen at age 12 to 18 months vs. those who did not were eight times more likely to have ASD when all children were considered and nearly 21 times more likely to have ASD when limiting cases to children with regression in development. Ibuprofen use at age 12 to 18 months was not significantly associated with ASD for either of these groups.

In a later report we showed that the events in the history of acetaminophen use were related to the number of children with ASD [19]. In this report, acetaminophen history was linked with the number of eligible persons with autism by birth year from a 1999 report to the legislature by the California Department of Developmental Services. We showed that the number of children with ASD greatly increased after the CDC issued a warning against using aspirin for children’s fever in 1980. We further showed separate decreases in the number of children with ASD born after highly publicized incidents in 1982 and 1986 where acetaminophen capsules were laced with cyanide in the US. These incidents caused precipitous declines in acetaminophen sales.

To continue investigating whether ASD is associated with acetaminophen use, we searched for available data which described acetaminophen use in older children with and without ASD. In 2015, we discovered data to re-test our ASD-acetaminophen link in children. The current study was undertaken to determine if acetaminophen use for fever in older children was associated with ASD.

## Materials and Methods

Our data source was the National Database for Autism Research (NDAR) of the National Institute of Mental Health (NIMH) which is one of the institutes in the National Institutes of Health (NIH) in the US. Approval for use of this data was obtained from NIMH and the University of Texas Health Science Center San Antonio. The data we chose to use was from the study entitled “Association between pupillary light reflex and sensory behaviors in children with autism spectrum disorders.” This de-identified data contained information on ASD diagnoses and over-the-counter medications used to treat fever. The selection criteria for these children may be found in the 2013 paper by Daluwatte et al. [20].

The diagnosis of ASD in our data was listed as confirmed by testing the children with the Autism Diagnostic Observation Schedule (ADOS) or the Autism Diagnostic Interview-Revised (ADI-R). In order to focus on the effect of environmental exposures, only children with ASD and without known genetic disease were included as cases. Genetic diseases linked to ASD which were excluded include fragile X syndrome, Down Syndrome, tuberous sclerosis, Rett Syndrome, and known ASD-linked chromosome insertions and deletions. After these exclusions, we obtained information concerning 155 children with ASD and 154 non-ASD children. We further limited our data analysis to the individuals for whom information regarding acetaminophen use for fevers was available which yielded 118 case children with ASD and 79 control children.

SPSS version 23 for Windows was used for all statistical analyses unless otherwise noted. Chi square tests were used to determine whether fever medication use was associated with ASD. Age at time of interview was tested for association with ASD by ANOVA. Logistic regression modeling was used to produce age-adjusted models for levels of fever medication use in children with ASD compared to children without ASD.

## Results

Table 1 shows the characteristics of children in our study. We limited our dataset to children with information regarding acetaminophen use for fevers. However, some of the other questions analyzed were answered by fewer respondents. Therefore for clarity, the number of cases and controls available for analysis of each question is listed in the table. There was no significant difference in the ages of the children, mean 131 months for cases and 135 months for controls. Acetaminophen use for fevers was significantly different for cases compared to controls (p = 0.013). Ibuprofen use for fevers was not significantly different between cases and controls (p = 0.570). Aspirin use for fevers was extremely low in both cases and controls; a total of four children were reported to ever use aspirin, and no comparative analysis was possible for aspirin use. Compared to control children, the frequency of fevers showed a trend for increasing rate in case children (p = 0.057), and case children were significantly more likely to show better social interaction when experiencing a fever (p = 0.037).

Table 2 shows the results of age-adjusted logistic regression models for three levels of acetaminophen and ibuprofen use compared to never or rarely use in case children with ASD vs. control children without ASD. Using acetaminophen as a first choice was 83% less likely in children with ASD (OR 0.165, 95% CI 0.045, 0.599) while use of acetaminophen if other medication doesn’t bring down fever was 82% less likely in children with ASD (OR 0.183, 95% CI 0.050, 0.675). Using only acetaminophen for fever showed a non-significant negative trend with a p value of 0.065. There was no significant difference in the three levels of ibuprofen use between cases and controls.

Table 3 shows the use of ibuprofen if acetaminophen doesn’t bring down fever vs. rarely or never use ibuprofen for children with ASD vs. non-ASD children while limiting the analysis to children who use acetaminophen as first choice. A significantly higher number of children with ASD vs. non-ASD children used ibuprofen to bring down their fever vs. rarely or never using ibuprofen (p = 0.033). This result indicates that significantly more children with ASD vs non-ASD children switch to the use of ibuprofen if acetaminophen does not bring down their fever.

## Discussion

As seen in Table 1, the use of acetaminophen in children with ASD compared to control children was still significantly different in the current study as it was in our 2008 study. However, as seen in Table 2, the association direction seen in our current study for acetaminophen use is opposite to our previous results which asked about acetaminophen use in young children. In our current study, older children with ASD compared to control children were significantly less likely to use acetaminophen for fever; whereas, in our 2008 study, younger children with ASD compared to control children were significantly more likely to use acetaminophen at 12–18 months of age and after the MMR vaccination. If we consider that early use of acetaminophen may be responsible for endocannabinoid system dysfunction, this could result in acetaminophen losing effectiveness. In this case, the results we found in older children are to be expected.

We have shown that the acetaminophen metabolites AM404 and p-aminophenol are toxic for mouse embryonic cortical neurons [21]. AM404 increases brain endocannabinoid levels by decreasing the re-uptake of anandamide [15]. We have further shown that acetaminophen differentially changes social behavior in adult male black and tan brachury tufted (BTBR) mice, a commonly used mouse model of behavioral traits of autism [22]. Neonatal exposure to acetaminophen affects cognitive function and reduces its analgesic and anxiolytic response in adult male mice and in our lab produced long-lasting immune system changes [23–25].

As we saw in our 2008 study, the use of ibuprofen in the current study is not significantly different for children with ASD vs. non-ASD children, although the use of ibuprofen has now increased from 56% to 87%. The children in our current study are older on average than our first study, 11 years vs. 7.5 years, and parents would have time to switch to a different anti-pyretic drug for their children if acetaminophen is no longer effective. We have shown in Table 3 that children with ASD vs. non-ASD children are significantly (p = 0.033) more likely to switch to the use of ibuprofen if acetaminophen doesn’t bring down their fever. The reasons for this reversal in analgesic use seen in our current study for children with ASD vs. non-ASD children could be a decrease in acetaminophen effectiveness due to endocannabinoid system dysfunction.

The endocannabinoid system plays a key role in the development of the central nervous system and its activation can induce long-lasting functional alterations [26]. Use of the exogenous cannabinoid tetrahydrocannabinol in the still-maturing brain may produce persistent alterations in brain structure and cognition [27]. Animal models have revealed the danger of both cannabis abuse and exposure to cannabinoid drugs during brain development [28,29].

Dysfunction of the endocannabinoid system can occur through either of the two classic cannabinoid receptors, CB1 or CB2. CB1 receptors are primarily located in the central nervous system (CNS) and are concentrated in the cerebellum, hippocampus, and the basal ganglia which are areas in the brain implicated as dysfunctional in ASD [30,31]. It has been demonstrated that during fetal life, CB1 receptors and their associated endocannabinoids provide axon guidance cues and are responsible for synaptogenesis [32–34]. Children with ASD have been shown to have abnormal brain connectivity which could be due to lack of CB1 axon guidance [7].

CB2 receptors are primarily located on immune system cells and serve a regulatory function. CB2 receptors have been shown to control the movement of inflammatory cells to the site of injury, and CB2 receptors’ reverse agonists may serve as immune system modulators [35]. CB2 receptor agonists reduce transendothelial migration of monocytes by interfering with endothelial adhesion [36]. It has been shown in many studies that children with ASD have immune system dysregulation [37–43]. This dysregulation includes differential monocyte responses, abnormal cytokine levels, decreased T cell mitogen response, decreased numbers of lymphocytes, and abnormal serum immunoglobulin levels. Other studies have shown that children with ASD exhibit abnormal antibodies against brain and central nervous system proteins [44–50] and increased plasma pro-inflammatory cytokine levels [51]. These problems could be due to dysregulation of the immune system through CB2 receptors.

Siniscalco et al. in a 2013 landmark paper were able to confirm endocannabinoid system dysfunction in ASD vs healthy subjects by showing in peripheral blood mononuclear cells that the mRNA and protein for CB2 was significantly increased and mRNA for the gene that synthesizes anandamide, N-acylphosphatidyl-ethanolamine-hydrolyzing phospholipase D (NAPE-PLD), was significantly decreased [52]. This upregulation of CB2 receptors and downregulation of NAPE-PLD in these immune system cells indicates endocannabinoid system dysfunction in children with ASD. This dysfunction could result from insufficient endocannabinoid system tone. Also in 2013, Földy et al. found that neuroligin-3 mutations associated with autism commonly disrupt tonic endocannabinoid signaling, providing further evidence of endocannabinoid system involvement in ASD [53].

The endocannabinoid system participates in fever generation by increasing anandamide activation of CB1 receptors [54,55]. We have shown in Table 1 that fever is associated with a significant increase in social interaction in children with ASD compared to non-ASD children. The increase in social interaction seen in this study could be due to fever producing a normalization of endocannabinoid tone in a system that has been made dysfunctional by acetaminophen use in children with ASD.

Kerr et al. have demonstrated dysfunction in the endocannabinoid system in the rat valproic acid model of autism. Alterations include reduced expression in the frontal cortex of PPARα and GPR55 and in the hippocampus of PPARγ and GPR55, which are additional receptor targets of the endocannabinoids. They found increased tissue levels of anandamide and palmitoylethanolamide (PEA) in the hippocampus after social interaction [56]. PEA is found abundantly in the central nervous system, especially in neurons and glial cells, and is a dietary supplement available without a prescription in Europe [57]. Administration of PEA has been shown to increase tissue levels of anandamide and increase activation of the endocannabinoid system [58]. PEA attenuates seizures in rats through CB1 and CB2 cannabinoid receptors [59]. Antonucci and colleagues in 2015 reported two case studies of boys with ASD in which PEA supplementation reduced inflammatory markers and produced rapid clinically significant improvements. With the success of these cases, they have recommended appropriate double-blind controlled clinical trials to further explore the potential of PEA as a treatment for ASD [60].

The warning in 1980 by the CDC against aspirin use in children has been very effective. As physicians began recommending acetaminophen as an alternative antipyretic drug, sales of children’s aspirin precipitously declined beginning in 1980 and were replaced with sales of acetaminophen [61]. As seen in our current study only four children were ever given aspirin for fever. However, since the initial CDC warning against the use of aspirin for fever in children, reports have been published casting doubt on the initial studies which associate children’s use of aspirin with Reye Syndrome [62–64]. As reported in an excellent review by Schrör in 2007, the attribution of Reye Syndrome to aspirin use in children was not sufficiently supported by research [65,66]. We have shown that the current rise in cases of autism began in 1980 in the US, which is the same year that the CDC warned against the use of aspirin in US children [19]. There is no good evidence that acetaminophen is superior to aspirin for use in children, and we have shown evidence that acetaminophen use is associated with ASD. We recommend that the use of acetaminophen in children be reviewed for safety. Also, the strong warning against the use of aspirin for fever in children should be reviewed.

## Conclusion

In summary, we have presented evidence for the association of acetaminophen use with ASD. Our theory of how this may occur can be explained in the following illustration. Suppose a susceptible young boy has a fever due to a viral infection or after the MMR vaccination. His parents give him acetaminophen which increases endocannabinoid stimulation in his brain making him feel better and bringing down his fever. But the increased activation of the endocannabinoid system also decreases immune system function which prolongs the illness and leads to even more acetaminophen use. Eventually, the boy recovers but his endocannabinoid system has been dysregulated to a lower level to compensate for the prolonged over-activation. Now the neurons in his brain are not getting the proper guidance for their growth through CB1 receptors and further suffer from increased inflammation due to lack of CB2 regulation in immune system cells. The boy develops ASD. When the boy gets a fever, his parents again give him acetaminophen but it no longer works well since his endocannabinoid tone is at a low level, and his parents switch to ibuprofen. Also, when he gets a fever, the increased anandamide levels briefly increase endocannabinoid tone and improve his sociability. After the fever, the endocannabinoid tone again drops back to low levels and his sociability decreases again. His condition, however, may be reversible with new cannabinoid medications to increase endocannabinoid system activation and allow his brain to slowly recover. Research needs to be conducted to see if PEA, cannabidiol, or other cannabinoids will be effective treatments for ASD.

## Figures and Tables

**Table 1:** Characteristics of children in the 2015 ASD -acetaminophen study by case-control status.

Variable	Cases	Controls	p value*
n = Cases, Controls	Mean (SD)	Mean (SD)	
Age (months) (n = 118, 79)	131.3 (43.6)	134.9 (39.6)	0.563^1^
	% (n)	% (n)	
Acetaminophen use for fever (n = 118, 79)			0.013^2^
Only use this	15.3 (18)	12.7 (10)	
First choice	35.6 (42)	46.8 (37)	
Use if other medication doesn’t bring down fever	31.4 (37)	36.7 (29)	
Rarely or never use	17.8 (21)	3.8 (3)	
Ibuprofen use for fever (n = 110, 73)			0.57
Only use this	9.1 (10)	4.1 (3)	
First choice	44.5 (49)	50.7 (37)	
Use if other medication doesn’t bring down fever	33.6 (37)	32.8 (24)	
Rarely or never use	12.7 (14)	12.3 (9)	
Aspirin use for fever (n = 90, 51)			1.000^2^
Only use this	1.1 (1)	0.0 (0)	
First choice	0.0 (0)	0.0 (0)	
Use if other medication doesn’t bring down fever	2.2 (2)	2.0 (1)	
Rarely or never use	96.7 (87)	98.0 (50)	
Frequency of Fevers (n = 117, 78)			0.057
Rarely (< 4 times / year)	85.5 (100)	93.6 (73)	
Sometimes (5–8 times / year)	10.3 (12)	6.4 (5)	
Often (>12 times / year)	4.3 (5)	0.0 (0)	
Social Interaction Better with Fever (n = 118, 79)			0.037^2^
No	81.4 (96)	92.4 (73)	
Yes	18.6 (22)	7.6 (6)	

* p values by Pearson chi square unless otherwise noted. A p value of less than 0.05 was considered significant and is marked in bold.

1 F test p value.

2 Fisher’s exact test p value, two sided.

**Table 2:** Age adjusted associations of acetaminophen or ibuprofen use for fever in children with ASD compared to control children by logistic regression.

Variable	B	Odds Ratio	95% Confidence Interval	p value*
Acetaminophen use for fever compared to rarely or never use:				
Only use this	− 1.352	0.259	0.062, 1.088	0.065
First choice	− 1.804	0.165	0.045, 0.599	0.006
Use if other medication doesn’t bring down fever	− 1.697	0.183	0.050, 0.675	0.011
Ibuprofen use for fever compared to rarely or never use:				
Only use this	0.782	2.186	0.467, 10.235	0.321
First choice	−0.16	0.852	0.333, 2.183	0.739
Use if other medication doesn’t bring down fever	0.001	1.001	0.374, 2.679	0.998

* p value by logistic regression likelihood ratio with a value less than 0.05 considered significant and listed in bold.

**Table 3:** Use of ibuprofen if acetaminophen doesn’t bring down fever vs. rarely or never use ibuprofen for children with or without autism spectrum disorder while limiting the analysis to children who use acetaminophen as first choice.

	For children who use acetaminophen as first choice:		
	Use of ibuprofen if acetaminophen doesn’t bring down fever	Rarely or Never use ibuprofen	p value* 0.033
Children with ASD	33	0	
Children without ASD	22	4	

* p value by mid-P exact test, OpenEpi Open Source Epidemiologic Statistics for Public Health, Version 3.03a
